# Histopathologic findings of tularemia lymphadenitis

**DOI:** 10.1007/s12308-026-00687-5

**Published:** 2026-03-03

**Authors:** Anna Dar, Ritu Banerjee, Emily F. Mason

**Affiliations:** 1 Department of Pathology, Microbiology, and Immunology, Division of Hematopathology, Vanderbilt University Medical Center, 445 Great Circle Road, Nashville, TN 37228 USA; 2 Department of Pediatrics, Division of Pediatric Infectious Diseases, Monroe Carell Jr. Children’s Hospital at Vanderbilt, Nashville, TN USA

**Keywords:** Tularemia, Necrotizing granuloma, Lymphadenitis

## Abstract

Tularemia is a rare zoonotic infection most often acquired through exposure to infected animals, arthropods, or contaminated food or water. Diagnosis typically involves serologic or PCR testing, but histopathologic findings can be a clue to the diagnosis. Here, we present a case of inguinal lymphadenopathy in an adolescent patient with a history of multiple animal exposures and possible tick bite. Excisional lymph node biopsy showed necrotizing granulomatous inflammation, and the clinical history, histologic findings, and serologic results together led to the diagnosis of ulceroglandular tularemia. This report adds to the limited available literature on the histopathologic findings of tularemia lymphadenitis and discusses the importance of including this entity in the differential diagnosis for necrotizing granulomatous disease.

## Introduction

Tularemia is a rare zoonotic disease caused by *Francisella tularensis*, a highly infectious gram-negative coccobacillus that is endemic to North America. In the United States, approximately 200 cases are reported annually, with the highest incidence in the south-central part of the country [1]. Data from the Centers for Disease Control and Prevention (CDC) suggest a recent increase in the incidence of tularemia, with most cases reported in children and older male patients [2].

*F. tularensis* is transmitted by direct contact with infected animals (such as rabbits, rodents, and domestic cats), drinking contaminated water, inhalation of contaminated aerosols, or via arthropod bite [3]. In the United States, arthropods that can transmit infection to humans include the dog tick (*Dermacentor variabilis*), the wood tick (*Dermacentor andersoni*), the lone star tick (*Amblyomma americanum*), and deer flies (*Chrysops spp.*). Infection is typically associated with fever and lymphadenopathy, with additional clinical manifestations dependent on the route of infection [4]. Ulceroglandular tularemia, the most common form, results from arthropod bite or contact through an open skin wound and causes an ulcer at the site of infection with associated regional adenopathy. Glandular tularemia presents with lymphadenopathy, without evidence of a skin ulcer. The oropharyngeal and oculoglandular forms are associated with infection via ingestion and exposure to the eye, respectively, while pneumonic tularemia occurs through inhalation. Finally, typhoidal tularemia is associated with fulminant systemic infection and a high mortality rate.

Tularemia is typically diagnosed based on clinical findings and history of potential exposure, with confirmation via serologic testing or polymerase chain reaction (PCR) [5, 6]. However, pathologists may encounter lymph node biopsies from patients with tularemia, which may be undertreated with empiric antibiotic regimens. When encountered in the laboratory, *F. tularensis* poses significant risk to microbiology personnel through potential inhalational exposure. Therefore, it is essential for pathologists to recognize when clinicopathologic findings raise the differential of tularemia. Here, we present an example of classic clinical and lymph node histologic findings in a patient ultimately confirmed to have tularemia.

## Clinical history

A 15-year-old male presented with right inguinal painful swelling. Approximately one month prior to presentation, he developed fevers up to 102.6 degrees Fahrenheit along with headaches, drenching night sweats, and posterior cervical lymphadenopathy. At that time, he was noted to have a lesion on his ankle thought to be caused by a tick bite. He was empirically treated with a 1-week course of doxycycline; testing for tick-borne infections was not performed. The patient’s symptoms initially improved. However, two weeks prior to presentation, he noted a raised, firm, painful area in the right groin as well as recurrent fevers, night sweats, and fatigue. An ultrasound revealed a 4.3 cm lymph node conglomerate in the right inguinal region. Testing for *Bartonella henselae*, *Histoplasma*, and *Blastomyces* was negative. He was treated with a 10-day course of amoxicillin/clavulanic acid with minimal improvement.

**Fig. 1:**
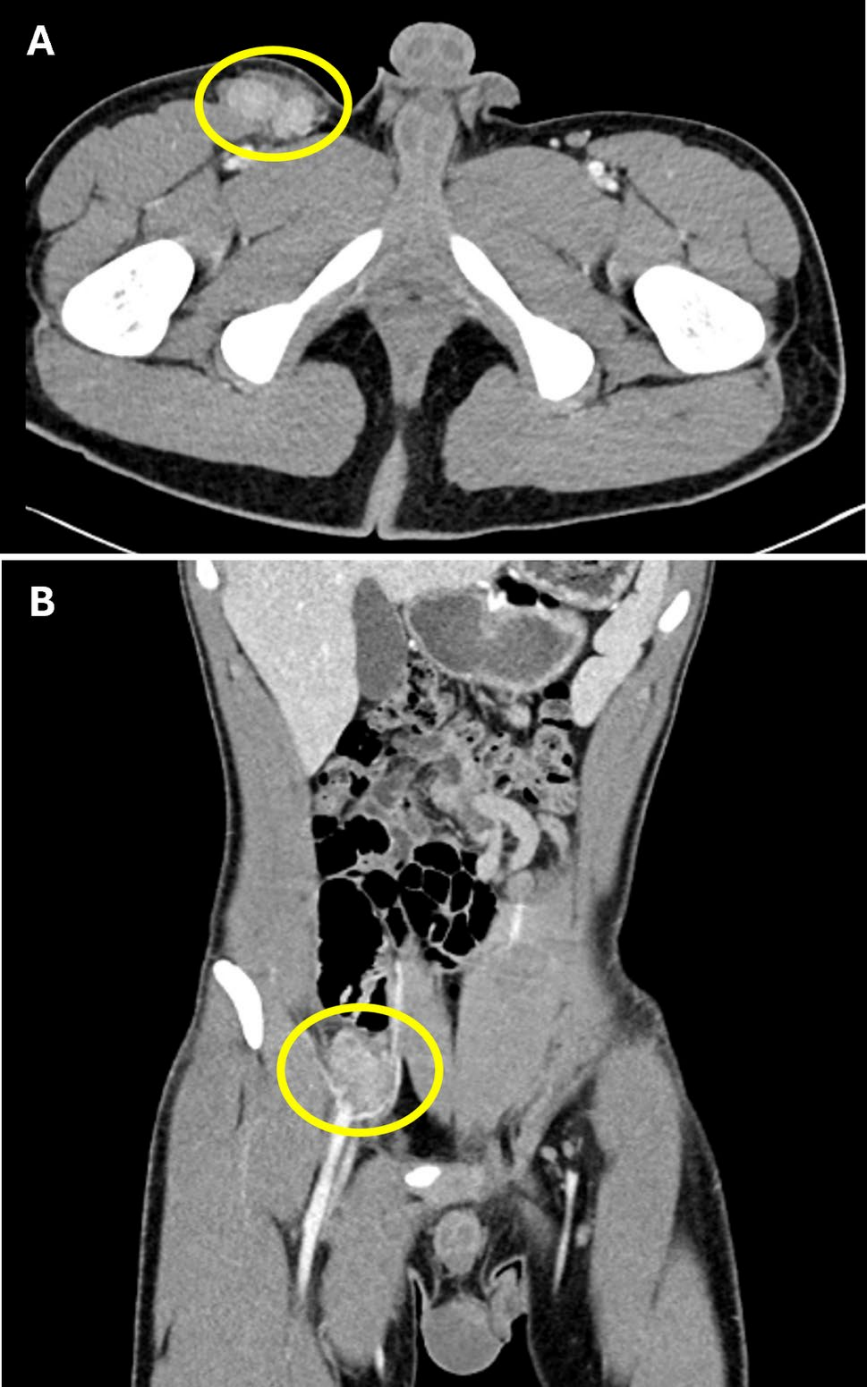
Multifocal lymphadenopathy on computed tomography (CT) scan. Axial (**A**) and coronal (**B**) CT scan images show enhancing enlarged lymph nodes in the inguinal (**A**) and external iliac (**B**) regions, highlighted by yellow circles

The patient reported multiple potential infectious exposures, through farmwork and exposure to pond water and numerous animals, including dogs, chickens, turkeys, ducks, pigs, and sheep, as well as multiple mosquito and tick bites. At the time of presentation to our institution, physical examination revealed large (> 3 cm) lymph nodes at the right upper thigh, very tender to palpation, with little mobility. A CT scan confirmed a collection of enlarged, hyper-enhancing lymph nodes in the right groin, as well as enlarged lymph nodes in the right external iliac, pelvic sidewall, and common iliac areas (Fig. 1A-B). Laboratory values were notable for elevated white blood cell count to 17.3 × 10^3^/uL (reference 3.4–10.2 × 10^3^/uL) with absolute neutrophilia (13.2 10^3^/uL; reference 1.8–7.7 × 10^3^/uL) and increased C-reactive protein (66.8 mg/L; reference 0–5 mg/L) and sedimentation rate (47 mm/hr; reference 1–33 mm/hr). Given the presence of multifocal lymphadenopathy unresponsive to antibiotic therapy, a right inguinal lymph node excision was performed, with tissue sent for histologic evaluation and microbiology studies.

## Results and clinical course

**Fig. 2:**
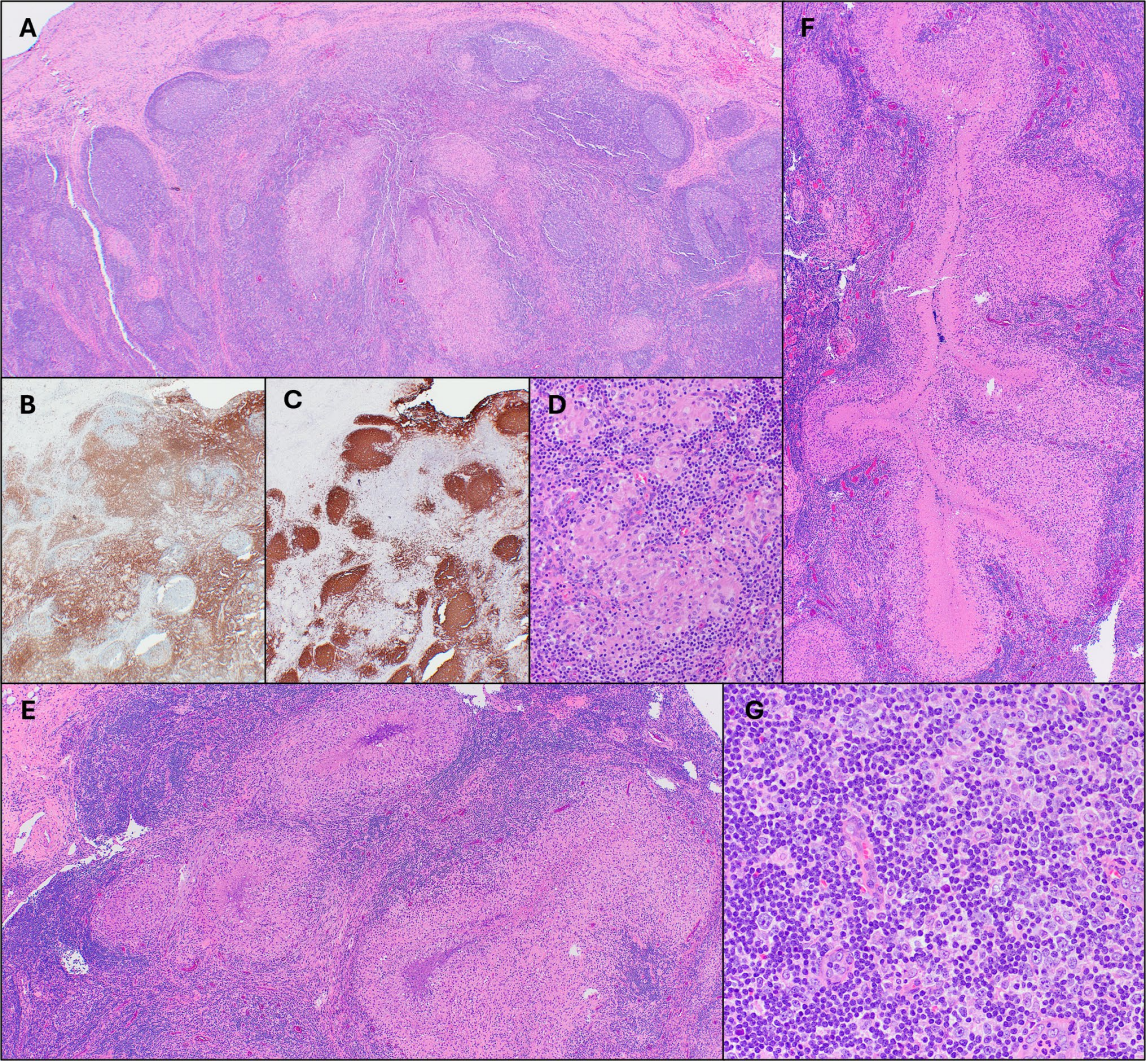
Histopathologic findings of tularemia lymphadenitis. The lymph node showed areas of follicular hyperplasia with variably sized reactive germinal centers (**A**; H&E, 2x). CD3 (**B**) and CD20 (**C**) immunohistochemical stains confirmed overall intact immunoarchitecture (both 2x). Numerous granulomas were present, ranging from small clusters of histiocytes (**D**; H&E, 20x) to larger irregularly shaped (**E**; H&E,10x) to serpiginous forms (**F**; H&E, 2x) with palisading histiocytes and extensive central necrosis. Scattered immunoblasts were present in paracortical areas (**G**; H&E, 20x)

Histologic evaluation of the excised lymph node demonstrated areas of follicular hyperplasia, with follicles containing reactive germinal centers (Fig. 2A). A CD3 immunohistochemical stain highlighted T cells, predominantly within interfollicular areas, while a CD20 stain highlighted B cells, predominantly within follicles (Fig. 2B-C). In addition, there were numerous granulomas, which ranged in morphology from small clusters of histiocytes (Fig. 2D) to large, often stellate to serpiginous forms with palisading histiocytes and extensive central necrosis (Fig. 2E-F). Some granulomas contained increased neutrophils with microabscess formation, consistent with suppurative granulomas. Very rare multinucleated giant cells were present. In some areas, small aggregates of monocytoid B cells were present around the granulomata, but monocytoid B cells were not prominent. Paracortical areas showed scattered immunoblasts (Fig. 2G), which were variably positive for CD30 and negative for CD15. No large or abnormal Reed-Sternberg-like cells were identified. In situ hybridization for EBV-encoded RNA (EBER) was negative. GMS and AFB special stains were negative for fungal and mycobacterial organisms, respectively. Flow cytometry performed on a portion of the lymph node showed polytypic B cells and mixed T-cell subsets.

The pathologic finding of suppurative necrotizing granulomatous inflammation was consistent with an infectious process. The microbiology laboratory was notified of the potential for multiple infectious agents. Concurrent serologic testing was negative for antibodies to *Histoplasma, Blastomyces, Toxoplasma, Rickettsia rickettsii*, and *Ehrlichia chaffeensis;* PCR for *Coxiella burnettii* was also negative. However, serology for *F. tularensis* demonstrated the presence of both IgM and IgG antibodies, suggestive of recent infection. Fungal and aerobic and anaerobic bacterial cultures from the lymph node remained negative.

In retrospect, the reported ‘lesion’ on the patient’s ankle following a tick bite was thought to represent a cutaneous ulcer associated with *F. tularensis* infection, and the patient was diagnosed with tularemia, likely ulceroglandular type. He completed a two-week course of ciprofloxacin and experienced resolution of all symptoms. At last follow-up, one month after presentation to our institution, the patient was clinically well, without infection recurrence or other complication.

## Discussion

Although perhaps best recognized as a Category A potential bioterrorism agent, due to the very low infectious dose, ease of spread, stability in the environment, and high mortality rate if untreated [1, 6], *F. tularensis* is an environmental organism endemic to North America. As such, sporadic infections occur, particularly in children, through exposure to infected animals and arthropod bites. Here, we present a patient with classic clinical and histopathologic findings of tularemia, likely acquired through a tick bite. The patient’s lymph node excision showed granulomatous inflammation in various stages, from small clusters of epithelioid histiocytes to large, serpiginous granulomas with palisading histiocytes, central necrosis, and suppuration. Our report adds to the relatively limited descriptions of the histology of tularemia lymphadenitis in the accessible recent literature. A large study by Lampset al. [6] from 2004 described histologic patterns seen in 16 cases of tularemia, but the study cohort was comprised predominantly of autopsy cases, including animal autopsies, and was therefore biased toward severe cases. Indeed, the authors reported that lymph nodes in their series were involved by necrosis, with only rare samples showing granulomatous inflammation. In contrast, a cohort of 54 lymph node samples described by Asanoet al. in 2012 [7] included no fatal cases and described progressive histologic changes, with follicular hyperplasia and small abscesses seen in the first 2 weeks after infection. Over 6 weeks, these lesions progressed to small epithelioid granulomas with central necrosis and finally to large necrotizing granulomas, which fused to form irregular shapes. In all stages, aggregates of monocytoid B cells were noted adjacent to granulomas. In the case reported here, the time from infection to lymph node excision was approximately 6 weeks, and pathologic review showed all stages described by Asano et al. within the same lymph node. An extended time between infection and diagnosis of tularemia has been reported, due, at least in part, to the nonspecific presentation and rarity of the disease [8, 9].

The histologic finding of necrotizing granulomatous lymphadenitis raises the differential of various infectious processes, particularly *Bartonella henselae* lymphadenitis (“cat-scratch disease”), *Mycobacterium tuberculosis* or atypical mycobacteria infection, and fungal infection, as well as *Chlamydia trachomatis* (lymphogranuloma venereum) and *Yersinia* infection in certain clinicopathologic settings [10, 11]. Exclusion of malignancy, including classic Hodgkin lymphoma, is also imperative. Indeed, as discussed below, tularemia may present as lymphadenopathy unresponsive to antibiotic therapy, raising particular concern for lymphoma.

*F. tularensis* is a fastidious organism that is difficult to culture in the laboratory. Diagnosis most commonly involves serologic testing and/or PCR-based detection of bacterial DNA. Nevertheless, lymph node or other tissue samples may be sent for microbiologic culture as part of the diagnostic work up to evaluate for possible infectious processes. *F. tularensis* poses significant infection risk to laboratory personnel, due to the potential for inhalational exposure and the low number of organisms required to cause infection [12]. Indeed, literature suggests that *F. tularensis* is one of the most commonly reported laboratory-acquired infections, although the total number of cases reported in recent decades is small [12, 13]. If a diagnosis of tularemia is under consideration, the microbiology laboratory should be notified so that laboratory personnel can take appropriate precautions when handling tissue specimens. This awareness will also allow the microbiology laboratory to incubate samples for extended time periods, which is necessary due to the fastidious nature of *F. tularensis*. Given the relative rarity of tularemia, this infection may not be included in the initial differential diagnosis of lymphadenopathy. Therefore, pathologists must be aware of the histologic pattern of tularemia lymphadenitis, in order to communicate the possibility of this diagnosis not only to the clinical team but also to laboratory personnel.

Inclusion of tularemia in the pathologic differential for necrotizing granulomatous lymphadenitis can have important therapeutic implications, as tularemia may be undertreated by empiric antibiotic regimens used for more common causes of infectious lymphadenopathy. Tularemia is not considered sensitive to beta-lactam antibiotics, and preferred treatment options include gentamicin or ciprofloxacin [14]. Doxycycline-resistance has been reported, and treatment with this drug has been associated with higher relapse rates [14, 15]; if doxycycline is used, a 21-day course is recommended. The patient described here initially received a 1-week course of doxycycline for a possible tick-borne infection and showed partial improvement in his symptoms. However, his lymphadenopathy ultimately progressed and systemic symptoms recurred due to inadequate antibiotic treatment.

The histopathologic finding of necrotizing granulomas is not specific and should raise consideration of infectious and neoplastic processes. The case presented here demonstrates the significance of acquiring a thorough clinical history pertaining to travel, occupational hazards (e.g., farmers, veterinarians, hunters, etc.), and other potential environmental exposures, not only to ensure accurate diagnosis and optimal therapy, but also to ensure the safety of laboratory personnel. We also add to the available recent literature regarding lymph node histologic findings in non-fatal tularemia. Ultimately, a multidisciplinary approach, involving correlation with clinical history, tissue histologic findings, and serologic studies led to the correct diagnosis and appropriate therapy for the patient’s disease.

## Data Availability

No datasets were generated or analysed during the current study.
